# Book Reviews

**Published:** 1814-07

**Authors:** 


### CRITICAL ANALYSIS
### *OF RECENT PUBLICATIONS*
IN THE
### DIFFERENT BRANCHES OF PHYSIC, SURGERY, AND MEDICAL PHILOSOPHY.

*Traite du Croup; par* M. Double. Paris, 1811.

WE are indebted for this work to an order issued by the late Emperor of the French, to institute an inquiry into the nature and cure of the Croup. The treatise is preceded by a preliminary essay on what the author calls monographies, or histories of single diseases, and on the best mode of drawing them up. It contains many useful remarks, but which have not sufficient novelty for insertion here. The author then proceeds to give a general history of the disease, which he divides into five stages. The symptoms of the first of these are stated to be fretfulness, increased heat of the skin, and other slight febrile affections: occasionally convulsions, bleeding from the nose, eruptions, and inclination to sleep. This is properly the period which precedes the attack of the disease, and is observed generally to occur towards the evening. The second stage, or accession of the disease, is marked by a cough, at first dry, afterwards attended with the expectoration of small mucous filaments; by a change in the voice, it becoming small, hoarse, or almost entirely suppressed; by pain in the throat; and mostly an inclination to sleep, from which the patient is awakened by a peculiar fit of coughing: but sometimes there is on the contrary insomnolescency. The countenance becomes flushed and tumid, and the eyes appear watery. This stage is generally observed to occur in the evening and during the

 night—

night—it is subject to irregular remissions, and the exacerbations commonly take place at the same period. In addition to these symptoms, during the third stage, or what M. Double calls the stage of erudity, the head is thrown backwards, breathing becomes difficult in the extreme, and is accompanied by an indescribable sound, which is very peculiar, and easily recognised when it has once been heard; sometimes this sound occurs only in coughing or in speaking: the cough brings on nausea and unsuccessful efforts to vomit; the urine is limpid, and a puffiness (bouffissure) of the scalp, thorax, and abdomen, is observed. During the two last-mentioned periods, a considerable remission of the symptoms is frequently perceived, but this is followed by an attack of increased violence. This fallacious remission is not attended by any critical evacuations, and occurs before the symptoms of the next stage have attained their height. The fourth stage is named by the author the stage of coction: it is distinguished by the evacuation of membranous concretions, by coughing or vomiting, by the urine becoming turbid and depositing a lateritious or whitish sediment, and by convulsive paroxysms of coughing. The return of the voice and respiration to their natural state, an intermitting pulse, without any alteration of the intellectual faculties, announce the approach of death. The fifth is the stage of convalescence: it is not observed to take place at any particular day of the disease, but may occur indiscriminately from the first to the twelfth, being regulated by the progress of the previous stages. An habitual cough, a tendency to inflammatory affections of the thorax, to phthisis, and to ulceration of the larynx (the phthisis laryngea of Portal), are among the sequelæ of croup.

The results of the examination of the bodies of those who have fallen victims to this disease, is the next subject upon which the author enters: he refutes an opinion which some have advanced, that suffocation is always the immediate cause of death in croup, by a case which fell under his own care, in which the patient experienced every symptom of croup; after death, the brain and its meninges, and the thoracic and abdominal viscera, were found in a healthy state; the interior surface of the larynx, and upper part of the trachea, was lined with a whitish thready substance, a little thicker than common pus, but not of sufficient consistency to offer any material obstruction to the passage of the air; no portion of the peculiar membranous concretion of croup was found on any part of the trachea. Ghisi and Home have also related cases of the fatal termination of croup before the membranous concretion had formed.

In

In the greater number of cases, M. Double has found the lungs in a healthy condition, but he has met with some instances of inflammation, of effusion of serum, and collection of pus in that viscus. If the bronchiæ be examined in their minute ramifications, they are mostly found loaded with mucus, but sometimes the mucous membrane is inflamed and dry. In the larger branches the mucus possesses more tenacity; it adheres to the sides of the trachea, where it forms a pseudo-membrane, varying very considerably in colour, consistence, and strength of adhesion. This order is now and then reversed, the fluid mucus being met with in the trachea and larynx, and the membrane in the ramifications of the bronchiæ. The internal mucous membrane of the trachea and bronchiæ is sometimes red, dry, and inflamed; sometimes whitish, tumefied, and moist; but most frequently retains its natural appearance. The mucous glands are more prominent than under ordinary circumstances.

The author, with laudable minuteness, proceeds, in three sections, to the examination of the anatomical, physical, and chemical properties of the membranous concretions of croup, the substance of which is, that they are infinitely variable with regard to size, figure, consistence, and colour, and he believes them to consist of the inspissated mucus of the parts. This last conclusion appears to us a little questionable. None of the author's chemical experiments lead us to doubt that the composition of these concretions is principally albumen; and we think their formation is much more satisfactorily accounted for, by supposing them to consist of a species of coagulable lymph thrown off by the increased action of the vessels of the parts, than by the author's conjectures. He made a number of experiments upon animals, by making them breathe atmospheres of chlorine (oxymuriatic acid gas), carbonic acid gas, nitrous gas, sulphureous acid gas, hydrogen gas, &c. diluted with various proportions of common air; and, upon examining the trachea, he generally found the mucus of a thicker consistence than ordinary; but he seems quite as much at a loss as we are to find out what useful inferences are to be drawn from these experiments. If people were never attacked with croup, unless they had been obliged to breathe artificial gases, such experiments might have thrown some light upon the subject; but in the actual state of the two cases, the total want of analogy between them must be evident. M. Double, we think, deceives himself in supposing, that, because the effects which sulphuretted hydrogen and carbonic acid gas produce upon the system are said to be sedative, these gases are therefore incapable of producing irritation or inflammation of the mucous membrane of the trachea. As far as our information extends, it appears most probable that all aeriform fluids which can be taken into the lungs, except atmospheric air, produce more or less irritation of the bronchial passages.

A disease very analogous to croup, has frequently been observed in animals. M. Double has collected many instances of this, and two epizootics fell under his own observation, one among cats, another among lambs, where the symptoms, as well as the appearances on dissection, had all the characters of this disease. Horned cattle, horses, pigs, and poultry, are occasionally liable to a similar affection.

We shall not attempt to follow the author through a long and elaborate analysis of almost every medical writer who has touched upon the subject of croup, from Hippocrates (whose pulmo repletus and angina gravissima he believes to be this disease) down to the present day, but shall content ourselves with taking a view of the general conclusions which he deduces from this examination. He does not consider croup as a new disease, but as one which has existed from the remotest periods: the diagnostic marks, however, have only recently been pointed out. He thinks that the cases of croup have not much increased in frequency, though more have been narrated lately, from the disease being more generally understood. It is met with in every climate, more particularly in places where the weather is cold and changeable. It is common in the northern parts of France. He denies the correctness of the observation, that it is peculiar to the sea coast. It is most frequent in the centre of the continent, and in some maritime situations is unknown. It appears in all seasons, but most commonly during cold and damp weather, or when sudden alternations from hot and cold take place. It most frequently attacks children between the periods of weaning and puberty; cases of it in children at the breast are very rare. In adults, it is still more uncommon, and the author hesitates to determine whether any of the recorded cases of supposed croup in them be instances of this or some other disease; but concludes that it has sometimes occurred. The number of each sex affected with this disease appears pretty nearly equal. There are no well-authenticated histories of croup which would induce us to consider it as having ever occurred as an epidemic, or that it is of a contagious nature. It is probable that a child who has once laboured under this disease is more liable to it afterwards when exposed to the exciting causes than another. Cases of croup are most frequently found to occur during a catarrhal epidemic, or during the

 prevalence prevalence of a gangrenous sore throat. M. Double believes that the relative size of the larynx and trachea in children is one cause of their being peculiarly liable to this disease, joined to the greater secreting activity of the mucous membrane, and the want of power to spit at an early age. This, however, is rather a lame mode of accounting for the fact. Several diseases, he says, leave a predisposition to croup, particularly measles, hooping-cough, catarrh, and inflammatory affections of the throat.

The author next gives us a chapter on the synonyms of croup, and on the different diseases to which authors have applied this term.* He proposes that croup shall be in future exclusively employed to indicate this disease.

We have a very able article on the diagnosis of this disease, through which we shall follow the author pretty minutely. The acute spasmodic asthma of children, described by Millar and Rush, is one of the diseases most commonly confounded with croup. The age at which these two diseases are common is the same, and they both occur during the prevalence of the same weather; they are both sporadic diseases, and have many symptoms in common. But in this species of asthma the cough is dry; there is no expectoration either of viscid matter, or of the peculiar membrane of croup; the voice is altered, but it is hollow and dull (sourd), and generally wants the peculiar character which distinguishes croup; the disease is not attended with fever; the patient can hardly breathe during the fit, except in the sitting posture, and whilst bending the head forwards; the fits recur every six, twelve, or twenty-four hours with well-

---

* Cœlius Aurelianus, Asthma. Baillou, Morbus incognitus, affectio orthopnoica. Const. Lyem. 1576, Lib. 2. Etmuller Catarrhus suffocans. Angina Maligna of Marcus Aur. Severinus & Bartholin. Maloccin Cynanche Maligna. Ghisi Angina Stupitosa. Some of Starr's cases of Morbus Strangulatorius. Van Bergen, Morb. Strang. A few of Wallbom's cases of Angina Aphthosa. Schultz Suffocation Striduleuse. Wilcke confounds croup and malignant sore throat under Angina Infantum. Home, Turnbull, Brooke, Underwood, Middleton, &c. Suffocatio Stridula. Michaelis, Angina polyposa seu membranacea. Salomon, Back, Morbus Strangulorius. Bard, Angina Suffocativa. Brera, Angina Membranacea. Chambon, Esquinancie Membraneuse ou Croup. Lentin, Croup mequeux. Croup by the generality of English and French writers. Engstroem, Angina Sufferatoria. The popular synonyms are, in the western parts of Scotland, the chock or stuffing; in some parts of England, the rising of the lights; in Sweden, Stryp-sjuka; and in Germany, die beaune häutige.

marked intermissions in the intervals. In croup, fever is present, the remissions are very imperfect, respiration is the same in every position of the body, but the patient throws his head backwards as if to lengthen the neck. The progress of croup is more rapid than that of spasmodic asthma. The author elucidates these observations by some cases of the spasmodic asthma. The disease which our author terms Catarrhus pulmonalis vel suffocans, we suppose another species of asthma, bears the closest analogy to croup. It is a rare disease in children, but not unusual among adults. It is unaccompanied by the peculiar sound of the voice, and by the membrane of croup, and its attacks are not preceded by the symptoms of irritation and pain about the trachea and larynx, which always announce the last-mentioned disease. The line of demarcation between croup and hooping-cough is distinctively marked. The alteration of the voice in this last disease is to a deep and sonorous tone, only observed during the convulsive inspiration which attends the fit of coughing. The patient is well except during these fits; is free from fever; and the constitution generally is not much affected. The membranous expectoration is wanting. The hooping-cough is attended with a sense of tingling about the præcordia; the croup with pain of the larynx and trachea. The hooping-cough is epidemic; the croup is not. The different species of quinsey, particularly cynanche maligna, have often been confounded with croup. It can only be at the commencement of the disease that any doubt on this subject can occur. Cynanche maligna is generally epidemic, and attacks children and adults equally; there is difficulty and pain in swallowing from the first, and inflammation and ulceration of the tonsils, pharynx, &c. which do not take place in croup. The voice is hoarse, but wants the peculiar character of the voice of croup. Cullen has described croup under the title of cynanche trachealis, but has evidently included under that name another and a very different disease, from which M. Double next distinguishes it. It is an inflammatory affection of the pharynx, larynx, and upper part of the trachea, principally affecting adults. In this disease, the pain is acute and shooting; in croup, obtuse, and rather a sense of uneasiness than pain; in cynanche trachealis the secretion of the mucous membrane ceases, and consequently little or no expectoration takes place; on dissection, it is found red and inflamed; the voice has not the character of croup; and the symptoms experience no remissions. It is not probable that a foreign body should find its way into the trachea without the knowledge of the patient; but, if this should be the case, the symptoms may be distinguished distinguished from those of croup by the suddenness of the accession, and the urgency of the disease, and by the absence of expectoration. Polypi in the trachea occasion but little pain, the symptoms come on very gradually, and the voice does not possess the character of croup. The points of resemblance between other diseases and croup are so few that they can give no difficulty to the practitioner.

The peculiar sound of the voice appears, then, to be one of the most characteristic marks of this disease, but still it is by no means sufficiently certain to be entirely depended upon. Home records a case of extraneous body in the trachea, in which the voice of croup was very well marked; and the same sound occurs sometimes in the spasmodic asthma, and the affection of the larynx which accompanies hydrophobia. Cases of croup have been met with and have proved fatal without this symptom. The sound itself is not sufficiently well marked and invariable to serve for a diagnostic symptom. It varies considerably with the degree of obstruction which the air meets with in its passage through the trachea, and with the part where the obstruction is situated. Neither is the membranous concretion to be considered as an infallible pathognomic sign of croup, as the author has collected some cases where it occurred in diseases of a widely different character, and some cases of croup, of which one or two terminated fatally, when the membrane was neither expectorated, nor could any traces of it be found on dissection.

We do not pretend exactly to comprehend our author's pathological ideas; nor can we take upon us to explain what he means by a catarrhal or nervous state of the constitution, nor by these *states* having a tendency to attack the trachea. Passing over some speculations on these subjects, we come to the practical division of croup into three distinct species, the catarrhal, the inflammatory, and the nervous. The first of these comes on with coryza and the general symptoms of catarrh well marked, followed, during the progress of the disease, by the expulsion of large quantities of mucous matter. The pain felt is obtuse; there is no tumefaction nor redness internally or externally; a quantity of mucous matter is generally present in the posterior part of the mouth. This is the species which is by far the most frequent, and which particularly depends on wet and cold weather: its progress is less rapid than that of the other species; the remissions are less distinct than in nervous croup, but more so than in the inflammatory species, and a marked exacerbation always comes on towards evening. The eyes are languid and humid, the countenance pale, perspiration considerable, and the expectoration of mucous matter and membranous concretions cretions abundant. The inflammatory croup is next in frequency: its attack is more sudden; the pain is more acute and more violent, and is accompanied with a sense of heat; the dyspnœa, and indeed all the symptoms are more urgent; the voice presents a modification which it is impossible to describe; the face is red and inflamed; the remissions are less distinct; the progress of the disease is more rapid; and favourable terminations are more frequent in this than in the other species. As this species is most frequently observed in individuals of a robust habit, the patients usually possess sufficient power to expel the membranous concretions. The favourable termination is ordinarily preceded by sweats, and the lateritious sediment in the urine. The most prominent symptom of the nervous species is the tendency in the exacerbations to observe a certain period, with regular intermissions. The cough is drier, the oppression and agitation are more intense, the pulse is small and wiry, and convulsions, or other marks of the derangement of the nervous system, are occasionally present. On dissection, the membranous concretion is found dry, and free from mucus, and the air cells of the lungs are full of air. This species is very rare, and hardly ever met with except combined with one of the other species, or with some other disease.

The author next considers the combinations of these three species with one another, and with other diseases, with somewhat frivolous minuteness. All these combinations of diseases present only a combination of their respective symptoms, with the exception of small-pox and scarlatina, in which the periods of the eruption are considerably modified by the croup, appearing slower and later, and disappearing sooner than under ordinary circumstances.

It appears from the chapter on the prognosis in croup, that two thirds of the cases on record have terminated fatally. The prognosis may be favourable if the practitioner be consulted before the third or fourth day of the disease, if the pulse be then strong, though frequent, and if the change of voice be only observable when the patient coughs or cries, and the respiration not extremely difficult. The symptoms which will strengthen this prognosis as the disease advances, are a free expectoration, and undiminished energy of the vital powers. The more distinct the different periods of the disease, and the older the patient, the greater will be the chances of recovery. The cases in which the progress of the disease is rapid, more generally terminate favourably than protracted cases. A considerable degree of irritability of the trachea, joined with an unexhausted state of the vital powers, furnishes a good indication. If called in late in the disease, disease, if the pulse be feeble, and there be much general debility, with laborious respiration and frequent fits of coughing, the practitioner will give an unfavourable prognostic. A frequent, irregular, and intermitting pulse, particularly when accompanied with a sudden return of apparently natural voice and respiration, is a very bad symptom. The appearance of the sediment in the urine can only be considered favourable when accompanied by other symptoms of the decline of the disease.

The author has allotted a very inconsiderable portion of his work to the discussion of the prevention and cure of the disease. He justly observes that in this, as in many other diseases, the cases and circumstances frequently vary so much, that a precisely-similar plan of treatment can hardly be appropriate to two separate cases; and that the judgment of the practitioner must always accommodate the remedies to the individual disease which he has to treat. M. Double's practice may be, and we believe is, very good, but some of the reasons which he assigns for it, and his ideas respecting the operation of certain medicines, are at best very whimsical. In catarrhal croup, he recommends repeated emetics, particularly ipecacuanha, for the purpose of giving an increased degree of sensibility to the trachea, to assist in the expulsion of the mucous and membranous concretions. Stimulant liniments and blisters to the neck and other parts of the body, dry cupping and irritating clysters, are all to answer the same useful purpose. Carbonate of ammonia and of potash are also serviceable. Calomel would be an excellent remedy in this species, *and that because it is a moderate stimulant of the lymphatic system and mucous membranes*, if its action was not too slow to counteract the rapid progress of the disease. Seneka root is also of some utility in this species of the disease only, from its irritating action. Bleeding is injurious. The author has not found sulphuret of potash to succeed in the cases in which he has given it a trial. Bleeding, general and by leeches, is the appropriate mode of combating the inflammatory croup. Refrigerants, as nitre and simple oxymel, and gentle laxatives, internally, with emollient poultices and fomentations to the throat, should be had recourse to. Emetics do no good. In nervous croup, M. Double enumerates the whole class of antispasmodics, assafœtida, musk, camphor, opium, æther, castor, hemlock, amber, oxyde of lime, &c. Some cases have appeared to derive much benefit from the liberal use of milk. As M. Double before recommended stimulant liniments to increase the irritability of the trachea, &c. so he would now employ them to take off the spasm of the the same parts. The inhalation of vapour, medicated with æther, opium, or cicuta, and pediluvia rendered a little irritating by the addition of mustard, are also said to be serviceable. In the complication of croup with small-pox, calomel is a medicine on which great reliance is to be placed, and is particularly to be given where there is any reason to apprehend that croup may supervene towards the termination of variola. Little is said with respect to prevention, except that exposure to the exciting causes is, of course, to be avoided.

The author concludes with some observations on the operation of tracheotomy, as recommended for the relief of croup. If, as he asserts, though we think without sufficient proof, the obstruction to the passage of air is *never* the cause of death, nor even of the most urgent symptoms, nothing further need be said upon the subject. But, as this is a point which we think by no means fully ascertained, we cannot at present join with him in this conclusion.

---

*Expériences sur le Principe de la Vie, notamment sur celui des mouvemens du Cœur, et sur le Siége de se Principe, suivies du Rapport fait à la Première Classe de l'Institut sur celles relatives aux mouvemens du Cœur.* Par M. LE GALLOIS, Doctor en Medicine de la Faculté de Paris, &c. &c. pp. 36[illegible]. 8vo. Paris, 1812.

(*Concluded from vol.* 31, *p.* 425.)

HAVING ascertained in the preceding memoirs that life may be prolonged after the separation of the brain by substituting artificial for natural respiration, our author next proceeds to enquire what is the longest possible duration of life under these circumstances, and what are the causes by which death is ultimately produced. Setting aside the incidental causes which may have some share in producing this effect, such as hæmorrhage, &c. which must be considered as resulting from the bad success of the means employed for the preservation of life; there are two connexions by means of which the brain may exert an action upon the life of the trunk through the spinal marrow and the par vagum, and both these connexions are destroyed in decapitated animals. We have already seen, that the mechanical phenomena of respiration depend upon the integrity of the former of these; and M. le Gallois seems to come rather hastily to the conclusion, that in applying an artificial substitute for these phenomena after the division of the marrow, we remedy every defect arising from the loss of this connection; whilst he justly observes, that we cannot be supposed to supply the deficiency occasioned by the destruction of the latter.

His

His object, then, was to enquire into the effects produced by the loss of this latter medium of communication independent of any other injury. Before proceeding to the detail of his own experiments, he gives us a neat abstract of the opinions which other authors have entertained upon this question. Ruffus and Galen are the first writers to whom he refers; they only state that the effects produced by the division of the par vagum are drowsiness and loss of voice. Piccolhomini asserts, that it produces death, by putting a stop to the action of the heart. Riolan denied the truth of this opinion, and repeated the experiment and obtained a contrary result. Willis, who placed the vital principle of the organs of involuntary motion in the cerebellum, and imagined that these nerves formed the principal medium of communication, found, like other theorists, that experiments confirmed his preconceived ideas. The division of these nerves, he said, was followed by such irregularity of the motions of the heart, sooner or later, as to destroy the animal. He explained the difficulty that the effect was not more instantaneous and decisive, by supposing that the brain continued to exert some influence over the action of the heart through the great sympathetic nerves. A long list of authors who espoused different sides of the question, is given by M. le Gallois; some of whom added the division of the sympathetic nerves to that of the par vagum, and without perceiving that death was materially accelerated in consequence. It also frequently happened, that death was the immediate result of the division of the par vagum only. Some of these writers, among whom is Haller, looked for the cause of death to the stomach; and others again, considered the principal agency of the division to be upon the lungs.

Amongst others, Bichat performed this experiment, and particularly noted its effects upon respiration. It is amusing to observe how ingeniously he twists the simple facts to bring them to tally with his theoretical notions. After informing us that the animal continued to breathe with great difficulty till death took place, he labours to prove that these nerves possess no actual influence over the lungs. M. Dupuytien resumed the investigation, and from the results of several experiments on horses and dogs, was led to assign suffocation as the cause of death. He attributed the suffocation not to any mechanical obstruction to respiration, but to the loss of an imaginary nervous influence necessary to the action of oxygen upon the blood. It is not easy to perceive what could be his grounds for adopting this opinion. M. Dumas, of Montpellier, believed that the effect was a mere mechanical obstruction to respiration, but he did not point out the exact nature of the obstruction. M. Blainville's experiments led him to agree with Haller, that the stomach was the organ principally affected. M. Provençal ascertained, that, after the division, the changes in the air respired were not effected to the same extent as before. Such was the state of opinions upon this question, when M. le Gallois took up the enquiry.

M. le Gallois had ascertained, by some previous experiments, that the length of time during which animals support deprivation of air, diminishes as the animals grow older. He was therefore much surprised to find that a puppy two days old died in less than half an hour after the division of the par vagum, whilst adult dogs survived the operation some days. He accidentally discovered, that the division of the recurrent nerves in a puppy two days old produced death in the same short period. Hence he was led to conclude, that the effects resulting from the division of the par vagum in the neck are generally much complicated with the effects of the division of the recurrents. As the recurrents are principally distributed to the larynx, he conceived that the division acted by contracting the opening of the glottis, and thus preventing the free access of air into the bronchia: he brought this opinion to the test of experiment, by making an opening into the trachea in young animals almost expiring in consequence of the operation, and found that the symptoms were instantly alleviated. The result of numerous experiments on dogs, cats, rabbits, and guinea-pigs, was, at the age of a few days after birth—the two former species only survive the division of the recurrents about half an hour; guinea-pigs of the same age about an hour; and rabbits some hours. Even in the last species, at this early age, death is produced as speedily by the division of the recurrents as of the par vagum. We wish particularly to impress upon our readers the age of the animal which is the subject of the experiment; as we believe it has been owing to want of attention to this circumstance, that doubts have occurred respecting some of M. le Gallois's conclusions. He states, that dogs and cats are not killed by the division of the recurrents after they have attained the age of three months. This circumstance is attributed to the proportional dimensions of the glottis varying at different ages.

To obviate some objections which were raised against his method of accounting for death after the division of the recurrents, the author made the following experiments. In rabbits two months old, he dissected out the larynx from the the os hyoides and neighbouring parts without injuring its muscles or the recurrent nerves, and then drew it sufficiently towards the chest to shew the opening of the glottis. The glottis appeared round, or partially oval from above downwards, and enlarged and diminished synchronously with respiration. On dividing the recurrents or par vagum, the arytenoid cartilages approached one another, and the opening was much diminished. In dogs and cats the opening was nearly closed. On dividing the recurrent on one side, the arytenoid cartilage on that side was seen to fall in and remain motionless. He removed the larynx entirely with a portion of the trachea, into the divided extremity of which he introduced a syringe: the air driven out from the syringe readily escaped through the larynx; but, on moving the piston into the opposite direction, he experienced a resistance similar to what would have occurred from placing his finger upon the extremity of the syringe.

It has been supposed that these results contradict those obtained by M. Magendie in his experiments on deglutition, of which we gave some account in our last volume: but a little attention will shew us they are perfectly reconcileable. It must be recollected, that M. Magendie's experiments were avowedly made on the adult animal. The distribution of the recurrents is to the crico-arytenoidei muscles, whose exclusive operation is to enlarge the opening of the glottis, by removing the arytenoid cartilages from one another; and also to the thyro-arytenoidei, which will partly conduce to close the glottis anteriorly, by drawing the arytenoid cartilages forward. This last distribution explains the reason of the greater difficulty of deglutition in M. Magendie's experiments when the division of the recurrents was added to that of the superior laryngeal nerves. The morbid closing of the glottis in consequence of the paralysis of the first mentioned muscles in M. le Gallois's experiments on young animals, was, of course, a very different affection, and would be productive of different effects from its healthy constriction in the moment of deglutition.

It appears, then, that the effects of the division of the par vagum upon the viscera of the thorax and abdomen, are always more or less complicated with the effects of the division of the recurrents upon the larynx. M. le Gallois attempted to obviate these last effects by making a large opening into the trachea, though this was generally attended with accidents which materially interfered with the results. In the experiments which appeared least equivocal, he did not observe that the period which animals survived the division of the par vagum bore any proportion to

 their their age. The lungs, the stomach, and the heart, were all evidently affected; but none of them to such a degree t at their functions are immediately suspended, except perhaps the stomach. The affection of the heart is indicated by a loss of fulness and strength in the arterial system, and want of regularity; it however is never so considerable as to be the immediate cause of death. The derangement of the stomach is much more considerable; indeed, its functions appear to be completely annihilated: but, as the animal almost invariably dies at an earlier period than would have occurred from total inanition, the cause of death must be sought elsewhere. M. le Gallois has never observed that putrescent state of the aliment in these animals, which some authors have described, and considered as the immediate cause of death. The stomach has sometimes presented slight appearances of inflammation, but this has rarely occurred. The affection of the lungs is the most constant and most urgent of the symptoms produced by this operation; it goes on increasing, respiration becoming more and more laborious till the animal dies. The lungs are found tumid with blood, but in distinct patches, so that portions of them, when thrown into water, will sink to the bottom. The bronchia are mostly filled with a frothy reddish serum. These appearances are very different from those which are observed after strangulation; but are very similar to what is discovered in people who die of a protracted affection of the chest with debility, particularly peripneumonia notha. The length of time which animals survive the division of the par vagum varies exceedingly. The extreme periods in thirty-two rabbits of different ages were six hours and a quarter, and eighteen hours and a half.

After decapitation, when artificial inflation of the lungs was practised, the same appearances were observed in the lungs. The serous effusion and bloody tumefaction took place more rapidly. Five hours and a half was the longest period that he was able to preserve rabbits alive after this operation. The general shock given to the nervous system probably accelerates death.

This memoir is followed by the report of the Committee of the National Institute, and some directions for the performance of the experiments more generally detailed in the memoirs. Three short notices on the dentition of rabbits and guinea-pigs; on the period of gestation; and on the relaxation of the joinings of the pelvis in parturition, of the latter, conclude this interesting volume.

The prosecution of these enquiries has afforded M. le Gallois the opportunity of ascertaining one or two points in

 the the natural history of rabbits and guinea-pigs, hitherto unnoticed. These two species have only one set of teeth, which are formed at first of a somewhat pyramidal shape, and are constantly and gradually protruding from the alveolar cells, so that the diameter of the tooth increases, as the upper part is worn down; thus accommodating it to the increased developement of the jaw. Rabbits possess a sixth molaris on each side of the upper jaw which has escaped the observation of zoologists. He has ascertained the period of gestation of the guinea-pig to be sixty-five days. He also found that in parturition, the symphysis of the pubis in the last-mentioned animals, was sufficiently separated to admit of the introduction of one or two fingers between the two bones; the sacro-iliac symphysis were also relaxed. Without a provision of this sort, it would be impossible for the head of the fœtus to pass through the pelvis, as the diameter of the former is nearly double that of the latter. The re-union of the symphysis takes place a few days after parturition.

---

*Account of Baths, and of a Madeira-House, at Bristol: with a Drawing and Description of a Pulmometer; and Cases shewing its Utility in ascertaining the State of the Lungs in Diseases of the Chest.* By EDWARD KENTISH, M.D. Physician to the Bristol Dispensary, and to St. Peter's Hospital. Longman and Co. 8vo. pp. 118. 3s. 6d.

THE utility of baths of various kinds, in promoting health, administering to luxury, and remedying disease, has long been acknowledged. Their adoption in this country, however, has been extremely partial, and their encouragement insufficient, the natural consequence is, that they are upon a parsimonious inconvenient scale; those which are used for cleanliness or pleasure, are constructed with little attention to excellence of plan, or pleasantness of effect; and those more especially appropriated to the diseased are too generally in the hands of ignorant and unqualified persons, who occasion mischief by misapplying a powerful remedy, and excite disgust by advertising the extraordinary cures which they pretend to perform. We are gratified then in observing, in the treatise before us, a regular physician devoting a considerable portion of his time and attention to this, in our opinion, important and much neglected means of cure, and hope that he will be rewarded by finding the institution which he has organised, realise his sanguine expectations. Dr. Kentish, some time ago, attempted to interest the public in a plan for establishing what he terms a Madeira-house, at Clifton, for the accommodation of invalids. The prospectus was warm, and the views it unfolded pleasantly santly luxurious. The happy patient was to have been transported into a sort of fairy palace; without, indeed, was a variable atmosphere, and all the miseries of the English climate; but, within, the combined advantages of the steady climate of the south of France, and the genial climates of Naples or of Madeira. This Hygeian temple, which we think a finer name than a Madeira-house, was to contain more conveniences for the patient than he could possibly have in any other situation; and it was intended he should "avail himself in one spot, of all the scattered gifts of Nature, which the experience of ages has proved to be beneficial for the restoration of health." The calculation of expence for all these and many more delightful recreations and comforts, for which we refer to the prospectus, was only fifty thousand pounds, and the plan "was warmly espoused by a few zealous medical friends;" yet, *proh puder!* "it unfortunately happens that we live in a period when millions are lavished for the destruction of man, and a few thousands are with difficulty raised to benefit the species;" and the scheme was of necessity abandoned. Dr. Kentish therefore not meeting with the support he wished, has formed a private institution, which is under his immediate controul.

"The entrance into the baths is by folding doors, by a plain unornamented portico, which opens opposite to the east end of the Cathedral,—a large open area, very commodious for the access to the baths either by carriages, chairs, or on foot. The situation is extremely convenient, easily accessible to the inhabitants of Bristol, and to the visitors of the Hotwells and Clifton, who may have occasion to use the baths.

"The large room, in which the baths are placed, is thirty feet long, by twenty feet wide; the cieling is fifteen feet high; and the whole is lighted by a dome light from above.

"From the entrance at the portico, a few gentle and easy steps conduct into the servants' lobby, through which you pass into the bath room; this is divided by partitions, twelve feet high, into four small chambers, the remaining area forming a sort of waiting room,—large, well lighted, and provided with seats and a table, where the bathers may amuse themselves with a book until the bath is prepared; or, after the use of the bath, remain in a middle temperature, previously to exposing themselves to the air.

"In two corners of the room there are large reservoirs; they are placed close up to the cieling, and are inclosed by partitions; they contain each about four hundred gallons, the one of hot, the other of cold water, which is conducted by pipes into each of the bath rooms, terminating in the baths, which are made of copper, and japanned in such a manner as to imitate the verd antique marble. From each of the baths there is a waste pipe, which carries off the water, when done with in the bath—these unite into one common pipe, which conveys it through the water-closets of the house. Thus the bather sees sees the water drawn fresh from the reservoirs for his own use, and may see it run to waste, if he chuses. By these means the impossibility of having a bath which has been used, is complete. Adjoining one of the warm baths, there is a shower bath, containing twelve gallons, which is charged with hot or cold water, at the wish of the patient. Into this bath also are conveyed the hot or cold douches.

" The warm water reservoir is provided with a false bottom, about two inches from the real one; between these two bottoms, a current of strong steam is thrown by the means of a small boiler, placed in a room below the bath room; the water above the false bottom absorbs the heat of the vapour, condenses it into water, which falls back into the boiler, where it again receives a proportion of heat, and carries it back to the false bottom. By this process the water becomes heated in the reservoir to about 150 degrees of Fahrenheit's thermometer; it probably might be carried higher. This is as high as I have had it, and is much more than an adequate heat for any purpose of bathing. The main steam pipe which passes from the boiler to the reservoir, goes through the vapour-bath room, from whence the steam is drawn, in any manner which may be required; it is made to pass in any direction, and may be impregnated with any substances that might be desired. The various vapour douches, jets, and other modes of locally applying this power to different parts of the body, are so arranged, as to be under the guidance of the assistant. This combination of local with the general use of the vapour bath, is capable of producing effects, which nothing less than having been a witness to them myself could have induced me to believe.

" In case the warm reservoir should have been too freely used, and the temperature should not be found equal to the required degree, each warm bath is provided with the means of being heated separately. One has a double bottom, between which steam is conveyed, which imparts its heat to the water, and may be thus raised to any temperature. The other warm baths are provided with a steam pipe, which descends to the bottom of the bath, and steam is thrown into the water, on the same principle as Count Rumford fitted up an apparatus for Mr. Gott, of Leeds."

Dr. Kentish's mode of heating the waters is ingenious. It is to be regretted, that the powers of steam have hitherto been so much neglected in their application to various domestic purposes in which they might be directed. Some useful hints may be derived from our author's suggestions. Several pages are occupied with an account of natural and artificial thermal waters, from which we extract the following passages for the attention of our readers.

" During the time of the French monarchy, it was the custom to send the maimed and crippled soldiers to Bareges, with a view of restoring to them the use of those limbs which had suffered from the hardships of war. When the revolutionary war raged, the number of these honourable victims increased in such abundance, as to preclude the possibility of affording them relief at the medicated springs.

Chemistry,

Chemistry, which had more than once been the saviour of the republic, was again put into a state of requisition. The chemists analysed the mineral waters at their sources, and indicated the means by which these natural productions might be imitated by art. The inspectors of the armies, Doctors Heurteloup, Parmentier, and Des Genettes, gave directions to the military hospitals to use factitious baths. These were prepared accordingly, and used under the same circumstances as the waters at their sources would have been used. In the year 1807, the inspectors drew up a report of this practice, shewing the result in several of the military hospitals.

"In the hospital at Toulon, seventy patients were submitted to the trial of factitious waters. Thirty-seven were much relieved; twenty-three were perfectly cured; and the remaining ten were in such a state as to preclude the expectation of relief. The hospitals at Rennes and at Lisle were not so fortunate in their number of cures. But, on the comparative scale of one hundred patients, treated by factitious mineral waters, and the same number at the sources of the natural medicated springs, the success of the former was fully equal to that of the latter."

Dr. Kentish then proceeds to inquire into certain analogies which exist between plants and animals, and details the opinions of Bichat, in supposing a combination of the animal with the vegetable organization. The effects of climate on vegetables and animals are explained; and from the numerous interesting facts which he has collected, the author concludes,

"1. That there is a great analogy between plants and animals.

"2. That plants, and the organic part of animals, are, in many instances, influenced by the same agents.

"3. That plants are entirely dependent upon climate.

"4. That an artificial climate may be prepared for plants, which will enable us to have any plants we wish, in any climate.

"5. That animals, as well as plants, are influenced by climate.

"6. That animals, as well as plants, suffer deterioration, disease, and death, from sudden and great changes of climate.

"7. That the salutary influence of an artificial climate is proved by our success in keeping exotics.

"8. That it is probable equal benefit would accrue to animals by an artificial climate; it would secure those who come from a southern zone, and would impart the genial influence of a more southern clime to the delicate and valetudinary of our own climate, who, from delicacy of structure, may be regarded as exotics."

This leads to the description of the Madeira-house, or Conservatory, which we have no doubt is well adapted for the purposes for which it is designed. In the appendix, an instrument called Pulmometer is described; it is for the purpose of ascertaining the power and capacity of the lungs to receive the atmospheric air, and seems a useful contrivance; but our account of it would not be complete without the etching which accompanies accompanies it. Some cases are subjoined to prove the success of Dr. Kentish's plan.

We cannot doubt that, occasionally, benefit may be derived from the artificial life to which patients must be subjected in a hot-house; but we fear when once they enter the walls of the Conservatory, they incur the hazard of becoming, in constitution, like the exotics which form part of the beauties of the Hygeian temple.

---

*Medico-Chirurgical Transactions, published by the Medical and Chirurgical Society of London. Volume the Fourth.* 8vo. pp. 490.—Longman and Co. 1813.

(Continued from Vol. 31, p. 511.)

XII. *Observations on the Cataract.* By BENJAMIN TRAVERS, Esq. Demonstrator of Anatomy at Guy's Hospital, Surgeon to the Hon. East-India Company, and to the London Infirmary for Diseases of the Eye.*

IT is with much satisfaction that we find the name of Travers annexed to a paper on Cataract. The very extensive opportunities which that gentleman enjoys of making observations on diseases of the eye, and of contrasting the results of the different operations proposed for the removal of cataract, warrants us in raising our expectations of receiving much information from his remarks. The opinion of medical men respecting which operation is most eligible, is still so much agitated by doubts, and perplexed by controversies, that a careful and impartial record of the different results is still greatly to be desired. For, though the many publications on this interesting subject, which are already before the world, ought to have precluded the necessity for such a work, yet men engaged in altercations, and urged by motives of interest, are so apt to paint their favourite objects in too glaring colours, and either do not trace their failures at all, or place them so much in the shade, that they are obscured by the more prominent and dazzling figures in the foreground.

Mr. Travers commences with some observations on the probable causes of opacity, the most obvious of which is inflammation, which is manifested by accidental wounds penetrating the crystalline, and rendering that body opaque; and by inflammation of the membranes, induced by blows or other causes, being followed by cataract.

"A conformation of body favouring a determination of blood to the head," he considers as another cause of opacity

---

* Two articles immediately preceding this paper have been omitted in our Analysis by accident.

of the lens. This observation we think may be strengthened by the known fact of horses that draw with collars, which are often tight and press on the jugular veins, being particularly liable to blindness. He next observes that "a frequent exposure of the eye to the stimuli of heat and light in more than ordinary intensity, or the habitual vision of minute objects in a depending position of the head, by which an undue proportion of blood is thrown upon the organ, commonly induce opacity of the crystalline or of the retina; which in one species of amaurosis turns of a green yellow colour, and becomes distinctly visible." We admit that such causes are sufficient to produce an opacity of the crystalline, but we must hesitate in subscribing to their "commonly inducing" such an effect. The great power of accommodation in nerves is very remarkable, and it is a well-known fact that a person who has been long confined in a dark place, where only a few dim rays of light have been admitted, will be able to descry objects which would be perfectly invisible to another man who had been exposed to the full blaze of day, and vice versâ. By use, the eye, like any other organ, adapts itself to that degree of stimulus which is applied to it, and in time becomes as insensible to its intensity as under ordinary circumstances we are to the light of day. We should rather say that frequent and sudden alternations from light to darkness would be more injurious to the eye than the continued application of the same stimulus. The author proceeds with remarking that cataract frequently occurs in old people without any preceding inflammation. "Transparent parts (he says) obviously tend to become opaque in age, as may be instanced by the want of clearness of complexion in old persons, and the arcus senilis, as it is called, which is an opacity without inflammation encroaching upon the cornea. The very minute serous vessels of the crystalline run in the cellular substance which unites the lamellæ. This interstitial texture is probably absorbed in age, and the vessels may be gradually obliterated by compression; but this must be matter of conjecture." With this last observation we most cordially agree, and cannot refrain from lamenting that such vague and useless speculations should be mingled with real matter of fact. We decline making any further remarks on this passage, which has flowed with too much rapidity through the author's pen.

He next remarks that "Cataracts are formed in utero, and I have rarely observed in the subjects of congenital cataract other marks of deranged or defective organization: some other more subtle cause of opacity must, therefore, be admitted." We are glad to find no attempts to discover this subtle cause, which from the preceding paragraph we somewhat dreaded.

Here follows some remarks on the various situation of cataract, and the mode of distinguishing their nature, which we insert at length.

"The cataracts of new-born children and of aged persons exhibit very opposite appearances. In congenital cases the opacity most frequently appears in the central nucleus, the interior denser structure demonstrated in the healthy lens by Petit, and is either stationary, or enlarges equally and slowly in a circle. This nucleus is sometimes not bigger than a pin's head in the centre of the transparent lens; but more commonly it is of the size of the contracted pupil, so that the child habitually knits his brows, or screens his eyes with his hand, to obtain that state of the pupil which he finds necessary to his vision. The fluid and capsular cataracts are exceptions to this observation. It is well known that adult subjects of cataract see better in moderate than in strong light, but in a much less degree; for the opacity is in them more diffused, so as very faintly, if at all, to exhibit a nucleus; and a dilatation beyond a natural one, I mean that obtained by the belladonna, though it enlarges somewhat the field of light, seldom permits of vision. The opacity commonly appears of equal consistency from the origin of the complaint, and in its progress the light is shut out from the whole sphere of the pupil. The hard cataract affords a partial exception to this remark, in which the nucleus, though imperfectly defined, is generally to be distinguished.

"The opacity is sometimes simply capsular, which is known by the uniform nebulous tenuity of the opaque membrane stretched over the transparent lens, and rendered more distinct by the dark tint reflected from the choroides. The cataract appears to be prominent in the pupil, which is sometimes slightly irregular. In this case, which is considered to be an incipient state of the cataract, as by the consequent opacity or absorption of the lens it becomes more dense and distinct, the quantity of light admitted is considerable.

"More frequently the opacity is simply lenticular, which is known by the cataract appearing more dense, voluminous, and varied in its colour and texture, and in relation to the plane of the iris, deeper seated; by the circularity of the pupil, and the greater degree of blindness in the natural state of dilatation. The motions of the pupil being regulated by the quantity of light which is admitted to the retina, its size depends upon the texture and bulk of the opaque lens, i. e. a very dense cataract keeps it dilated by excluding light from the retina; a very bulky one by mechanically distending it. In most cases of congenital cataract, and in some of mature age, the dilatation by belladonna discovers a defined margin to the opacity, and a transparent circle beyond it, and therefore adds considerably to the patient's perception of light. I have known patients in this state, who were of an age to judge for themselves, decline the operation, content with the vision they enjoyed by the use of the belladonna. In such cases, however, a tolerable vision has been previously enjoyed, owing to the smallness of the opaque nucleus compared with the transparent portion of the lens. And in all cases the vision of near objects is confused, if not totally bedimmed, by the enlargement of the pupil with the belladonna, although that of distant ones is clear and distinct. Where a transparent circumference has been discovered after dilating the pupil by the belladonna, I have never seen the capsule opaque, and I believe this black rim may be considered as diagnostic of the transparency of the capsule. Where the lenticular opacity is diffused, this sign of a transparent capsule is of course wanting.

"The opacity is sometimes much deeper seated, so that you look at it through the transparent capsule and lens. It is here generally circumscribed, but irregularly shaped; and often, from its tenuity and depth of situation, escapes the observation even of oculists. This is usually considered to be a third seat of opacity, distinct from the former, viz. in the posterior covering of the lens. I do not find, upon repeated and strict examination, any proper capsule investing the lens, i. e. which admits of being removed with it. It may be necessary to a right understanding of this structure, briefly to describe it. The tunic of the vitreous humour advances to the ciliary body, there it separates into two laminæ, which, when contiguous to the margin of the crystalline, adhere closely to each other, forming the sacculated circle (canal godronné) described by Petit, which is capable of being inflated around the margin of the lens. This canal corresponds in breadth to the breadth of the ciliary processes, and is marked by them anteriorly. The anterior lamina, which is the more dense of the two, covers the crystalline in front; the posterior lines the fossula of the vitreous humour. There is no communication betwixt the canal of Petit, the vitreous humour, and the crystalline capsule, They are all distinct from each other, and must be inflated distinctly, if perfect. The crystalline, it will appear from this description, is incased in a duplicature of the vitreous capsule. The different texture of these laminæ adapted to their respective uses, (the one properly belonging to the crystalline, and supporting the whole lens in its place; the other proper to the vitreous, and covering a very small portion of the humour, which is sufficiently supported by the crystalline itself) and likewise the close investiture of the margin of the lens, which interrupts continuity, for it prevents the passage of air, explain why they are so seldom similarly affected in disease. The posterior opacity before described is therefore seated in the proper tunic of the vitreous humour. Thus much on the situations of the opacity forming cataract.

"The varieties of consistency, colour, and figure, are numerous. With regard to consistency, we have the fluid or milky, the flocculent or fleecy, the caseous or doughy, and the compact or hard cataract. The fluid lens is, I believe, rarely contained in a transparent capsule. The latter, in my experience, has been partially opaque, presenting a dotted or mottled surface. The capsule appears in contiguity with the margin of the pupil, and as if projecting in it, and the opaque spots upon it are most disguishable when it is viewed laterally. The second usually resembles, in appearance, flakes of snow irregularly heaped, being visibly of a loose and broken texture, and the larger masses intersected by semi-transparent lines: the arrangement is sometimes regular and uniform, being either foliated or radiated.

radiated. The capsule is sometimes semi-opaque, but more frequently transparent. The third is the cataract of greatest bulk, impeding the motions of the pupil, having a heavy and dense appearance, uniformly opaque, a clouded not a fleecy whiteness, and sometimes a greenish or dirty white tinge. The fourth appears deep seated, of a brown yellow, or amber colour, most dense in the centre; if entirely opaque, flat upon the surface, over which the iris plays freely. The second and third species are most commonly met with; the first and fourth are comparatively rare."

These observations are very valuable, as the result of much experience; but we doubt if these nice distinctions can always be so clearly established as to enable a practitioner to say with perfect confidence what the exact consistency of a cataract is previous to an operation. When this can be done, it is of much importance; for, as Mr. Travers very judiciously observes, the operation should be selected according to the nature of the case.

"To the first and second cases," he says, "*formerly regarded as incurable on account of their softness*, the operation performed by the late Mr. Saunders is admirably adapted. To the two latter the operation of couching or extraction is best suited." We must pause here for a minute to inquire what meaning the author attaches to the word "formerly." Does he refer to a period some centuries distant? or, as his next sentence would seem to imply, does the formerly relate to the time antecedent to Mr. Saunders, to whom the author seems to give all the credit of a perfectly-original operation? In looking back to the writings of Celsus, we find the following lines, after a most concise but clear account of the mode of securing the patient, and all the previous stages of the operation of couching: "Ubi eo ventum est inclinanda acus ad ipsam suffusionem est, leniterque ibi verti et paulatim eam deducere infra regionem pupillæ debet; ubi deinde eam transiit vehementius imprimi, ut inferiori parte insidat. Si hæsit curatio expleta est. Si subinde redit *eadem acu magis concidenda, et in plures partes dissipanda est: quæ singulæ et facilius conduntur, et minus late officiunt.*" Thus, then, we find that, eighteen hundred years since, the operation of breaking down the substance of the opaque lens was practised by Celsus. The limits of a review will not allow of our prosecuting our enquiries further, nor of tracing the rise and progress of this operation; but we cannot in justice pass over the accurate description of the late Mr. Pott, of the very operation proposed by Mr. Saunders.

"When the opake crystalline is in a state of dissolution, or the cataract is what is called perfectly soft, if the capsula of it be freely wounded by the couching-needle, the contents will immediately issue forth, and, mixing with the aqueous humour, will render it more or less less turbid: sometimes so much as to conceal the point of the needle and the iris of the eye from the operator.

"This is a circumstance which has been observed by most operators, and has been mentioned by many writers: but it has always been regarded and mentioned as an unlucky one, and as being in some degree preventative of success; which is so far from being the fact, that as far as relates to this circumstance merely, all the benefit which can be derived from the most successful depression, or extraction, most frequently attends it; as I have often and often seen.

"The aqueous humour, however turbid it may become, will, in a very short space of time, be again perfectly clear; and, if no disorder of the capsula of the crystalline, previous or consequential, prevents, the rays of light* will pass without obstruction through the pupil,

* "The capsula, or investing membrane of the crystalline, has very often an unsuspected share in the apparent opacity of that body, and is thereby the cause of disappointments and inconveniences during some operations, and after others. This is a circumstance which, undoubtedly, has been mentioned; but has not been by any means sufficiently attended to. The capsula is capable of becoming white and opake, while its contents shall be clear and transparent; it becomes so sometimes by being wounded by the couching-needle, used either for the depression of a firm cataract, or for the letting out a soft one; and it will not infrequently be found so, after the operation of extraction, when no instrument has touched it.

"Whenever this happens, it is an unpleasant circumstance; but still more so if it continues for any length of time: I have seen it disappear in a week; I have seen it continue two, three, or four, and at last totally disappear; and I have seen it continue so long as to require the re-application of the instrument. When it appears after the depression of a firm crystalline, or after an unsuccessful attempt to depress one which has proved not firm enough, it may easily be, and generally is, mistaken for a portion of the cataract risen again; but from which an attentive observer will always be able to distinguish it. But, when such opacity follows what is called a successful extraction, in which the cornea only was divided, the capsula not touched by the instrument, and the cataract came away entire through the pupil, the case is self-evident.

"This may truly and properly be called, as it has been by Monsieur Houin, Haller, and others, a membranous cataract, as it consists merely of the membranous capsula of the crystalline.

"Writers of credit have mentioned, that a cataract may be formed almost instantaneously, by external violence. There is no doubt of the fact: I have seen it four different times.

"Whether this be not an affection of the capsula merely, I much doubt; or rather am much inclined to suspect that it most frequently is. In three of the four, which have fallen under my observation, the opacity has gradually disappeared after the inflammation, in consequence of the blow, had gone off, and the eyes were left as clear as ever—a consequence which, I think, may be accounted for, by supposing the opacity in the capsula only; but cannot, if we suppose it to be in the corpus crystallinum itself."

and and the patient will be restored to as perfect vision as could have followed the most successful operation of either, or of any kind in the same subject, and under the same circumstances.

"When the cataract is of the mixed kind, partly soft, and partly hard, the immediate effects of the needle are somewhat different; the soft part of the cataract being less in quantity as well as generally less soft, the aqueous humour is less turbid, and the firm part or parts of the crystalline will be very visible. In this state, these firmer parts will very frequently elude the attempts made by the needle to depress them, and will therefore remain in the posterior chamber. This is also reckoned among the unfortunate circumstances; but, although to an operator not aware of, nor acquainted with, the consequence, it may have all the appearance of being so, yet it really is not; the true end and aim of the operation not being thereby necessarily frustrated. In this case, if the needle has been so used as to have wounded the capsula very slightly, it will sometimes happen, that the firm part of the crystalline will remain in its nidus, and still form a cataract, which may possibly require a future or re-application of the instrument. This is the worst that can happen, and happens indeed very seldom; for if the capsula be properly wounded, so that the aqueous humour be freely let in, the firm part or parts, though very visible at first, and preventing the passage of light through the pupil, will in due time, in some longer, in others shorter, gradually dissolve, and at last totally disappear; leaving the eye as fair, as clear, and as fit for vision, as any the most successful operation could have rendered it; of which I have seen and exhibited many proofs.*

"In order to render the fact still more clear, I have sometimes, when I have found the cataract to be of the mixed kind, not attempted depression, but have contented myself with a free laceration of the

* "The space of time which the accomplishment of such dissolution will require, is very uncertain; I have seen the eye perfectly fair and clear within a week after the operation; and I have seen it require two months for the dissolution of all the opake parts.

"This has been observed by many, even before the nature and seat of a cataract were truly known; among the rest, by Read, who, speaking of one of his own operations, says,

"'At the end of nine days I visited my patient, and found both her and her friends highly discontented, so that I met with nothing but invectives, &c.

"'Within a fortnight after, when art and nature having performed their mutual operations, and all the cloudy vapours and rags of the cataract were consumed and dispersed, her eyes grew clear, and her sight became perfect, &c.

"'I would have every patient, though after a cataract be couched, and nine or ten days expired, he see little or nothing at all, or that he cannot endure the light for a month or two, or even for a quarter of a year, as I have known many, not to be discouraged; for their sight may, notwithstanding, become well and perfect, and continue so ever after. On the other hand, some come to good and perfect sight within a fortnight or three weeks.'"—Sir W. Read, p. 7.

capsula; capsula; and, having turned the needle round and round between my finger and thumb, within the body of the crystalline, have left all the parts in their natural situation; in which cases I have hardly ever known them fail of dissolving so entirely as not to leave the smallest vestige of a cataract.* In a few instances, where I have had fair opportunity, I have pushed the firm part through the pupil into the anterior chamber, where it has always gradually and perfectly dissolved and disappeared, not producing pain or trouble, while such dissolution was accomplishing." †

Let any impartial reader compare the above description with the operation proposed by Mr. Saunders, and he will be at a loss to say in what the latter gentleman's claims to novelty consist; except that he employed these means at a much earlier age than was recommended by Mr. Pott. We should be sorry to detract in the least from the reputation of the late Mr. Saunders, but cannot, as reviewers, permit such a gross plagiarism to pass unnoticed.

---

* "The operation of extraction, though said in general to remove the crystalline entire, and calculated for such purpose, does not always do so; but, when the cataract is of the mixed kind, does not infrequently leave some of the firmer part behind, which, one of the warmest patrons of the operation allows, does dissolve and disappear. 'Extrahendum statim post operationem est quicquid remanet opaci ope Cochlearis Davielis. Hoc quidem facile sit aliquando, aliquando vero et imprimis ubi membrana crystallina non satis lacerata cochlear in ipsam capsulam lentis, ubi hæret illud opacum corpusculum non admittit, tantis difficultatibus circumfusum est, ut quicquid etiam moliaris extrahere illud non possis, et ne oculum nimis irrites, desistere ab opere, et relinquere illud in oculo cogaris.

"'Neque tamen tunc etiam spe optimi successus destituimur. Sæpe enim observavi, opacum illud remanens, sive sit mucus, sive frustulum lentis crystallinæ, sensim, et sponte, citius vel tardius, penitus disparuisse. An resorbetur mucus lacteus, an frustula lentis crystallinæ liquescant sensim, et resorbentur, an in fundum oculi sensim, se præcipitant, dubium est. Utrumque tamen fieri credo. Quoties lactea materia post depressam cataractam totum humorem aqueum opacitate sua et albedine inficiens sensim penitus evanuit? Quoties pus in oculo hærens vel sanguis insigni quantitate in illum effusus, sensim resorptus evanuit? Quoties frustula lentis crystallinæ, post depressionem cataractæ, in pupillâ relicta? &c. immo liquescere aliquando et resorberi hæc frustula me ipsum experientia docuit,'" &c.—Richter de Cataractæ Extract.

† "I should be sorry to have it inferred from hence, that I would recommend the passing the opake crystalline through the pupil, far from it; I think it wrong, as it is apt to produce one of the most frequent inconveniences attending the operation of extraction—an irregularity of the pupil. I only meant to prove the fact of dissolution of the cataract in such situation; and that it will not cause that pain and trouble which it is so positively said to do."

There

There is an observation of Mr. Travers's, with regard to the fluid cataract, worth attending to; namely, that he has twice known severe inflammation arise from a "solid bed of lens, which had been contained in the fluid, being set at liberty by the laceration of the capsule and oppressing the iris." We do not find any other original matter in his observations respecting the fluid and soft cataracts. In firm and hard cataracts he candidly avows, that, after a trial of considerable length, he has ceased to employ the operation of Mr. Saunders, as he terms it. In this we most perfectly concur with the author, as we have long since foreseen the fate of this much-extolled operation for producing the absorption of the firm cataract *in situ*. The length of time requisite for a perfect restoration to sight, and the constant danger during this period of the lens falling forward and pressing on the iris, are sufficient reasons to deter us from ever attempting such an operation where the lens is firm and cannot be broken up by the needle.

The author abruptly breaks off at the most important and interesting part of the subject, and tells us, that in some future communication "he will point out the circumstances which should determine the election of couching or extraction in the two latter, *id est*, firm species of cataract." As this is the only interesting point on which we expected any elucidations, we cannot but feel disappointed with this distant prospect of what we expected to have met with in the present paper.

Before we conclude, we must make a few observations on an expression, of which we find Mr. Travers, in common with many other authors, makes frequent mention—we mean the solution of the opaque lens in the aqueous humour.

We are not aware of any experiments having been instituted to prove the solvent power of the aqueous humour; but, from what we know of its qualities, are much inclined to be sceptical on this subject.

As the operation formerly practised by Messrs. Travers and Saunders on the hard cataract, namely, the removing a portion of the anterior part of the capsule, and exposing the texture of the lens to the action of the aqueous humour, rest entirely on the supposed solvent power of this fluid, it will be as well briefly to consider what facts we are possessed of respecting its solutive qualities. To begin with the soft or milky cataracts: when the capsule is punctured the opaque fluid gushes out into the anterior chamber, and renders the aqueous humour turbid, which in a very short space of time will again become transparent. This at first sight does appear like solution, but, if we consider for one minute with what astonishing rapidity the aqueous humour is replenished after being evacuated, we must suppose that there is a very considerable state of activity both in the exhalents and absorbents of the anterior chamber and iris, as we cannot suppose so great an increase in the healthy actions of a part to be suddenly set up. Clearly then the absorbents remove the opaque milky fluid, in common with the aqueous humour, through which it is diffused, but not dissolved. The same may be observed with regard to the soft flocculent portions of the second species, which are passed through the pupil into the anterior chamber, and are there rapidly absorbed. They come in contact with the same absorbents which are constantly employed in removing the aqueous humour. Where then, in this second case, is the necessity for referring to the solvent power of the aqueous humour? But, say the advocates for solution, the lens will be absorbed *in situ* if you open its texture to the action of the aqueous humour. This appears very plausible; but in reply we would observe, that in congenital cataract, after a time, the fluid part is absorbed within its capsule, and nothing but the dense membrane remains. Here, then, the absorbents of the capsule have been able to effect the removal of the fluid lens without calling in the aid of the aqueous humour: so also, in very old firm cataracts, we find the lens much shrunk and smaller than natural, arising probably from the absorption of the more soft parts. When a firm cataract is depressed and buried in the vitreous humour, in process of time it is absorbed; yet we do not hear of the solvent powers of the vitreous humour, which certainly is as much entitled to that property as the aqueous. From what has been observed, we conceive that the removal of the opaque lens, either *in situ* or when dislocated, does not depend on any solvent power in the humours, but on the absorbents of the capsule being excited by the operation, when the cataract is left *in situ*; and, when it is broken up or dislocated, its removal depends on its being separated from its natural connexions, and thereby rendered a foreign body, and acted on accordingly by the surrounding absorbents.

(To be continued.)

